# Aging impairs bimanual isometric force smoothness

**DOI:** 10.17179/excli2026-9581

**Published:** 2026-09-02

**Authors:** Hajun Lee, Hanall Lee, Jinwon Shin, Eo-Jin Son, Nyeonju Kang

**Affiliations:** 1 Department of Human Movement Science, Incheon National University, Incheon, South Korea; 2 Division of Sport Science, Incheon National University, Incheon, South Korea; 3 Division of Health and Kinesiology, Incheon National University, Incheon, South Korea; 4 Division of Sport Science, Sport Science Institute & Health Promotion Center, Incheon National University, Incheon, South Korea

**Keywords:** older adults, force control, fine motor control, force variability, YANK

## Abstract

Older adults normally showed impaired fine motor control capabilities, as indicated by increased force error and variability during submaximal force control tasks. However, whether aging additionally disrupts temporal smoothness of the force trajectory remains unclear beyond the magnitude of force fluctuation. This study investigated age-related changes in bimanual isometric force smoothness using YANK analysis (i.e., time-derivative of force; d*F*/d*t*). Fifteen younger adults (mean ± SD of age = 25.4 ± 2.5 years) and fifteen older adults (mean ± SD of age = 64.9 ± 6.5 years) performed bimanual hand-grip isometric force control at 10 % of maximum voluntary contraction across the vision and no-vision conditions. Force accuracy (relative root-mean-square error; rRMSE), variability (coefficient of variation; CV), smoothness (YANK), and interlimb force coordination (correlation coefficient) were quantified. Receiver operating characteristic (ROC) curve and Pearson's correlation analyses were conducted to evaluate the discriminative ability of each variable and potential relationships between YANK and conventional force control variables. Older adults showed significantly higher YANK values than younger adults in both vision and no-vision conditions, indicating reduced force smoothness. Greater rRMSE, CV, and correlation coefficient values for older adults were observed only in the vision condition. The ROC curve analysis revealed that YANK effectively discriminated between the younger and older adults in both vision and no-vision conditions. For older adults, increased YANK was significantly correlated with greater rRMSE and CV in the vision condition and with greater CV in the no-vision condition, whereas no significant correlations appeared in younger adults. These findings suggest the YANK approach can capture age-related fine motor control deficits that conventional variability measures may overlook.

See also the graphical abstract(Fig. 1).

## Introduction

Maintaining fine motor control capabilities in elderly people is important for increasing independence in daily activities (Billot et al., 2020[5]; Shizuka et al., 2025[52]). However, aging progressively induces structural and functional changes in the neuromuscular system such as loss of motor units and increased variability in motor unit discharge rates (Hunter et al., 2016[27]; Seidler et al., 2010[50]). These neurophysiological alterations additionally interfere with abilities to accurately and steadily produce and regulate force outputs, leading to impairments in fine motor control in aging populations (Camacho‐Villa et al., 2025[8]; Marmon et al., 2011[42]). Considering successful force control is normally required for executing various motor actions (Pethick et al., 2022[45]; Qiu et al., 2025[47]), investigating altered force control patterns in older adults may provide insights into age-related neuromuscular adaptations (Camacho‐Villa et al., 2025[8]; Carr, 2024[9]; Lodha et al., 2016[40]; Vieluf et al., 2017[58]).

Importantly, bimanual force control capabilities are related to many activities of daily living such as buttoning a shirt, carrying objects, or tying shoelaces, contributing to functional independence in aging populations (Ballardini et al., 2019[1]; Rattanawan, 2022[49]). Successful bimanual force control typically requires precise interlimb force coordination indicating accurate modulation of the combined summation of forces from both hands to reach a single and shared target level (Hu and Newell, 2011[26]). To achieve this common goal, two hands continuously interact and compensate for each other's errors imposing higher neural demands on the supplementary motor area, dorsolateral prefrontal cortex, and interhemispheric communication through the corpus callosum (Goble et al., 2010[18]; Maes et al., 2017[41]). However, given that aging is accompanied by reduced neural efficiency and structural degeneration of the corpus callosum (Cabeza et al., 2002[7]; Delvenne and Malloy, 2025[13]), older adults presumably show impaired bimanual force control as compared with younger adults.

Previous studies investigated bimanual force control capabilities in older adults under both vision (i.e., continuous visual feedback on bimanual forces and a target level) and no-vision (i.e., withdrawal of visual feedback on bimanual forces) (Critchley et al., 2014[11]; Kang et al., 2019[31]; Kenway et al., 2016[32]). The modulation of visual information during bimanual force control tasks can provide meaningful information on age-related alterations in visuomotor processing and dependency on visual feedback for fine motor control. In fact, older adults revealed greater force variability with visual feedback as indicated by standard deviation (SD) and coefficient of variation (CV) of force production than younger adults in the vision condition (Galofaro et al., 2025[17]; Hu and Newell, 2011[25]; Ko and Kang, 2024[34]; Lin et al., 2019[38]). Moreover, the differences in force variability between older and younger groups were more evident in the vision condition than the no-vision condition at lower targeted level (Critchley et al., 2014[11]; Kenway et al., 2016[32]; Tracy et al., 2015[57]). These findings suggest that aging impairs visuomotor processing while modulating lower intensity of forces because of increased variability of the descending command to motor units (He et al., 2017[22]; Li et al., 2023[37]; Seidler et al., 2010[50]). However, these conventional variability variables primarily quantify the magnitude of fluctuations from the mean force, and may overlook smoothness of the force trajectory in time-series (Kim et al., 2026[33]; Yacoubi and Christou, 2024[60]).

Interestingly, Lin and colleagues suggested the use of change rate in force fluctuation (time-derivative of force; d*F*/d*t*), named YANK, for estimating temporal smoothness of forces (Lin et al., 2019[39]). This biomechanical variable can be used for capturing the dynamics of force generation and the response of the sensorimotor system (Soto and Cros, 2011[53]). Previous studies estimated movement smoothness by calculating jerk (i.e., time-derivative of acceleration; da/dt) or spectral arc length during upper limb movements such as reaching tasks (Bayle et al., 2023[3]; Gulde and Hermsdörfer, 2018[20]; Yan, 2000[61]). These findings indicated that aging increased movement intermittency, suggesting that older adults struggle to perform continuous and natural motor adjustments. Instead, their movements consist of excessive but less effective motor corrections because of neuromuscular changes (Djafari et al., 2025[14]). Similarly, for bimanual force control tasks, greater YANK values of force can denote abrupt motor corrections while either processing visual feedback (i.e., vision condition) or depending on proprioceptive feedback (i.e., no-vision condition) (Kim et al., 2026[33]). Thus, estimating force smoothness using the YANK analysis may provide additional information on altered fine motor control strategies and underlying neuromuscular mechanisms in older adults.

The purpose of this study was to investigate force smoothness of older adults using YANK analysis during isometric force control tasks. To specifically estimate fine motor control, participants executed bimanual hand-grip isometric force control tasks at 10 % of maximum voluntary contraction (MVC) across vision and no-vision conditions, respectively. The rationale for selecting 10 % of MVC as a targeted level is twofold. This level represents the low force range essential for daily fine motor tasks (Godde et al., 2018[19]; Strote et al., 2020[54]). Moreover, force control ability at the lower targeted level is sensitive to age-related neuromuscular alteration (Oomen and van Dieen, 2017[44]). Specifically, force production below 10 % of MVC relies on recruiting a small pool of low-threshold motor units and is primarily regulated by modulating their discharge rates (Hunter et al., 2016[27]). With aging, motor unit discharge rates become more variable (Christou, 2011[10]), and the surviving motor units are enlarged through denervation and reinnervation processes (Hepple and Rice, 2016[24]; Jang and Van Remmen, 2011[28]). Thus, focusing on force control at 10 % of MVC may provide an optimal window to capture these underlying age-related changes in force control (Oomen and van Dieen, 2017[44]). We quantified and compared bimanual force accuracy, bimanual force variability, bimanual force smoothness, and interlimb force coordination between older and younger adult groups. Further, the receiver operating characteristic (ROC) curve analysis was used for determining which force control outcome variable successfully discriminates age-related fine motor control characteristics from younger adults. Finally, we performed correlation analyses between YANK and other conventional force control outcome measures as well as interlimb force coordination for each group. Based on prior findings that aging increased discrete motor actions (Morrison et al., 2013[43]), we hypothesized that older adults would reveal more reduction of force smoothness across vision and no-vision conditions than those for younger adults.

## Methods

### Participants

Fifteen younger adults (5 females and 10 males; mean ± SD of age = 25.4 ± 2.5) and fifteen older adults (6 females and 9 males; mean ± SD of age = 64.9 ± 6.5) participated in this study. To determine the required sample size, we conducted an a priori power analysis using G*Power software (version 3.1.9.7) based on the interaction effect size obtained from pilot data (partial ƞ^2^ = 0.263) (Faul et al., 2007[16]). With an alpha level of 0.05 and a statistical power of 0.80, the analysis indicated that a minimum of 26 participants was required. Participants were recruited from the university and the local community. We included younger adults aged between 20 and 30 years and older adults aged over 60 years. Exclusion criteria were: (a) musculoskeletal deficits, (b) neurological disorder, (c) history of surgery, fracture, pain, and movement limitations in their upper limbs. We instructed participants to avoid excessive physical activities and substances such as alcohol, caffeine, and painkillers for 24 h before the experiment. Prior to the experiment, all participants read and signed a written informed consent with a detailed explanation of the study procedures approved by the Institutional Review Board of Incheon National University.

### Experimental procedures

**Figure 2:**
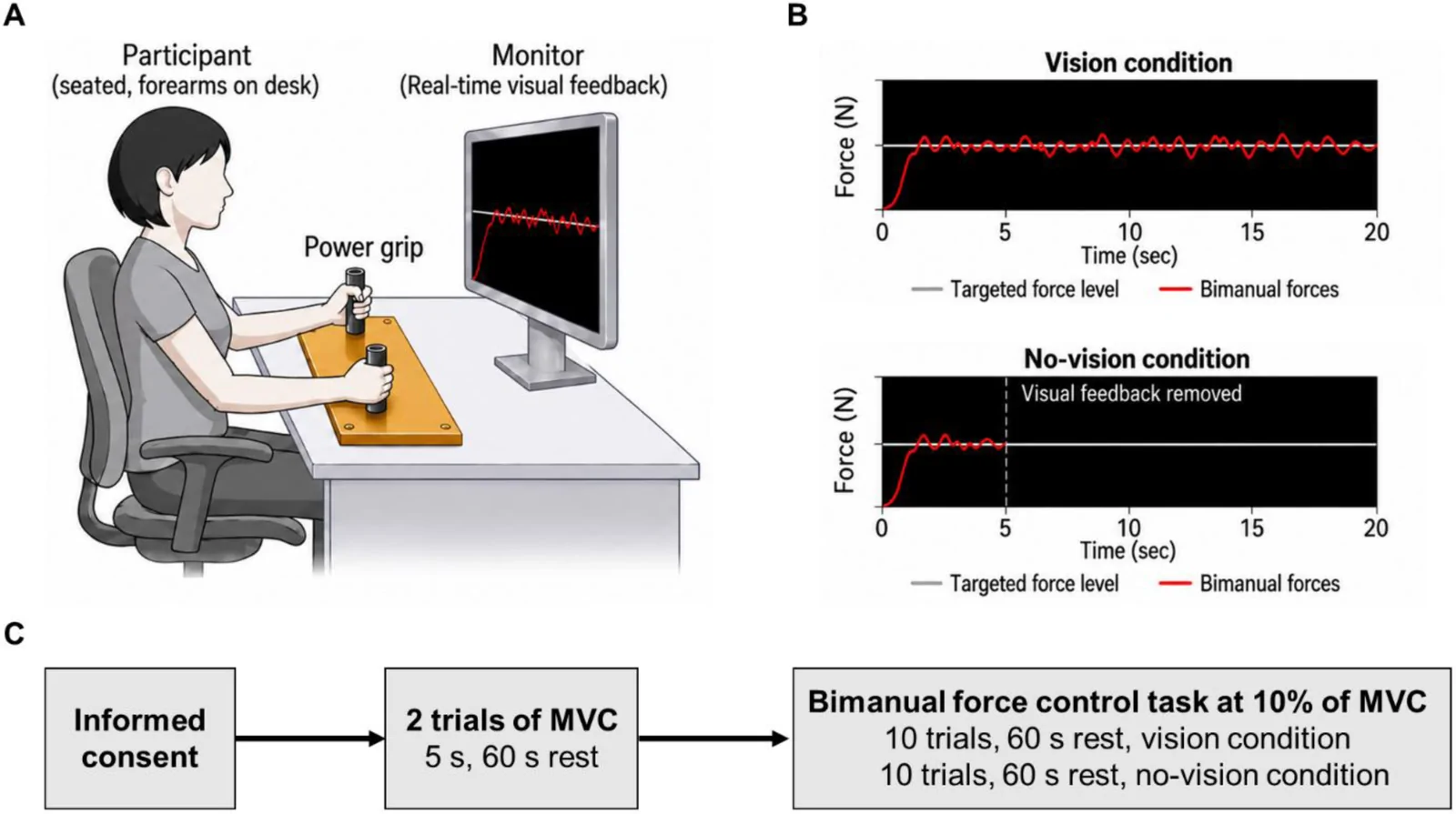
Experimental setup. (A) Bimanual isometric force control position. (B) Visual feedback in the vision and no-vision conditions during force control tasks. (C) Experimental procedure for bimanual isometric force control.

A customized bimanual isometric force measurement system (SEED TECH Co., Ltd., Bucheon, South Korea) was used. Participants sat 80 cm away from a 54.6-cm LCD monitor (1920 x 1080 pixels; 60 Hz refresh rate) and gripped the left and right handles (30 mm diameter) embedded with force transducers (Micro load Cell-CZL635-3135, range = 220 lbs., Phidgets Inc., Calgary, Canada) (Figure 2A(Fig. 2)). Participants maintained a comfortable position (i.e., 15°-20° of shoulder flexion and 20°-45° of elbow flexion) and fixed both arms to exclude unexpected force produced by movements of the elbow and shoulder. To determine the submaximal targeted force level, participants performed two MVC trials for 5 s with 60 s of rest between trials. The targeted force level was set at 10 % of the average peak force from the two MVC trials.

The goal of the bimanual isometric force control task was to produce and regulate the bimanual force around a targeted force level (i.e., 10 % of MVC) for 20 s. Participants performed submaximal force control tasks in both vision and no-vision conditions to investigate the potential effects of impaired visuomotor processing in older adults on YANK values (Kim et al., 2026[33]) (Figure 2B(Fig. 2)). We provided two types of visual feedback on the screen, including the red trajectory line (bimanual forces) and the white horizontal line (targeted force level). The vision condition provided continuous visual feedback of bimanual forces for 20 s. In contrast, the no-vision condition removed the visual feedback after the initial 5 s, requiring participants to maintain force without visual feedback for the remaining 15 s. Participants completed 10 trials with 60 s of rest between trials, and the order of the vision and no-vision conditions was randomized (Figure 2C(Fig. 2)).

All experiment procedures were conducted using a custom Microsoft Visual C++ program (Microsoft Corp., Redmond, USA). Data acquisition was performed at a sampling rate of 200 Hz using a 16-bit analog-to-digital converter (ADS1148 16-Bit 2kSPS) with a minimum detectable force of 0.0192 N. The force signals were amplified using an INA122 with 5 V excitation voltage (Texas Instruments Inc., Dallas, USA).

### Data analysis

All offline analyses were conducted using the Matlab program version R2024b (MathWorks TM Inc., Natick, MA, USA). For each trial, we focused on the middle 14 s of force data by eliminating the first 5 s and last 1 s to minimize initial adjustment and termination effects. Further, the force data was filtered by a bidirectional fourth-order Butterworth filter at a 30 Hz cut-off frequency. To assess bimanual isometric force control capabilities, we calculated the following variables: (a) mean force (average of bimanual force outputs), (b) force accuracy (relative root-mean-square error; rRMSE; root-mean-square error / target force × 100), (c) force variability (CV; SD of force output / mean force × 100), and (d) force smoothness (YANK).

Force smoothness was quantified using YANK, the first time derivative of force (d*F*/d*t*) (Lin et al., 2019[39]), capturing the change rate of force rather than the amplitude of force fluctuations (Kim et al., 2026[33]; Yacoubi and Christou, 2024[60]). YANK can be elevated by the greater level of absolute force, similar to the way movement jerk increases with higher movement amplitude (Sherman et al., 2024[51]). Producing a higher force requires the recruitment of more and larger motor units, which inherently induce greater force fluctuations (Enoka and Farina, 2021[15]). Consequently, this increases the raw YANK, even if the underlying smoothness of the force output remains unchanged. For this reason, Sherman and colleagues (2024) noted that absolute YANK values cannot be validly compared across groups producing varying force magnitudes (Sherman et al., 2024[51]). Supporting this, Yacoubi and Christou (2024[59]) recommended normalizing the metric to the mean of force, especially when comparing populations that exert equal effort but differ in maximal strength (Yacoubi and Christou, 2024[59]). Given that older adults in this study produced lower absolute forces than younger adults at 10 % of MVC because of their lower maximal strength, we normalized raw YANK to the mean force (Equation 1) to ensure that group differences reflected force smoothness rather than differences in absolute force production. Higher normalized YANK values indicate reduced force smoothness independent of force magnitude.


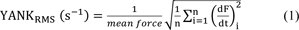


Where d*F*/d*t* represents the change rate of force at sample *i* and *n* denotes the total number of data points.

In addition, to evaluate interlimb force coordination, we calculated Pearson's correlation coefficient between the force outputs of the left and right hands (Hu and Newell, 2011[25][26]; Kang et al., 2019[31]). Positive correlation coefficients (0 < r ≤ 1) reflect an in-phase coordination pattern, with values closer to 1 indicating less effective interlimb force coordination. Negative correlation coefficients (−1 ≤ r < 0) represent an anti-phase coordination pattern, with values closer to -1 indicating more effective interlimb force coordination.

### Statistical analysis

An independent t-test was conducted to compare MVC values between younger and older groups. Additionally, we conducted a two-way mixed ANOVA (Group × Visual condition; 2 × 2) for the five force control outcome measures: (a) mean force, (b) rRMSE, (c) CV, (d) YANK, and (e) correlation coefficient. Post-hoc analyses were performed using Bonferroni's pairwise comparisons. ROC curve analysis was performed to evaluate the discriminative ability of each variable that showed significant differences between groups. Diagnostic accuracy was evaluated using the Area Under the Curve (AUC) classified as: (a) 1.00-0.90 = excellent, (b) 0.89-0.80 = good, (c) 0.79-0.70 = fair, (d) 0.69-0.60 = poor, and (e) 0.59-0.00 = fail (Hanley and McNeil, 1982[21]; Lasko et al., 2005[36]). Further, we performed Pearson's correlations between YANK and conventional force control outcome measures (i.e., rRMSE and CV) as well as interlimb force coordination (i.e., correlation coefficient) in the vision and no-vision conditions for each group. All statistical analyses were performed using IBM SPSS Statistics 28 (SPSS Inc., Chicago, IL, USA), with the alpha level set at 0.05.

## Results

### Bimanual maximal and submaximal force production

**Figure 3:**
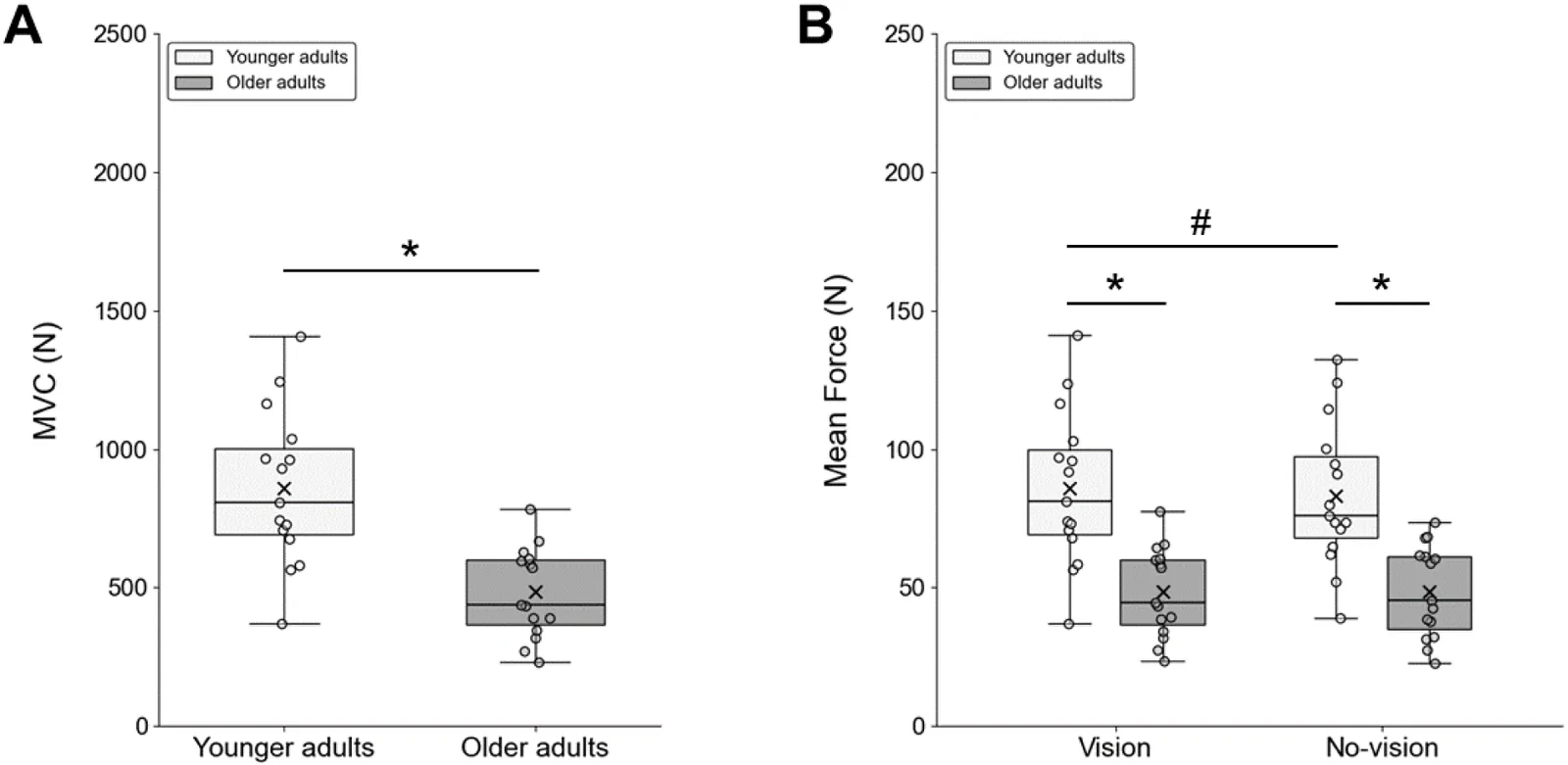
Bimanual maximal and submaximal force production. (A) MVC showing a significant difference between younger and older adults. (B) Mean force showing a significant Group × Visual Condition interaction. Asterisk (*) indicates a significant difference between age groups. Number sign (#) denotes a significant difference between visual conditions. P < 0.05.

An independent *t*-test on the MVC showed lower maximal forces in the older group than the younger group (*t*_28_ = 4.505; *P* < 0.001; Figure 3A(Fig. 3)). A two-way mixed measures ANOVA on the mean force revealed a significant Group × Visual Condition interaction [*F *(1, 28) = 5.385; *P* = 0.028; partial ƞ^2^ = 0.161; Figure 3B(Fig. 3)]. Post-hoc analyses showed that the older group produced lower submaximal forces than the younger group across vision (*P < *0.001) and no-vision conditions (*P < *0.001). Further, the younger group exhibited higher mean force in the vision condition than the no-vision condition (*P = *0.006).

### Bimanual force control performance and coordination

**Figure 4:**
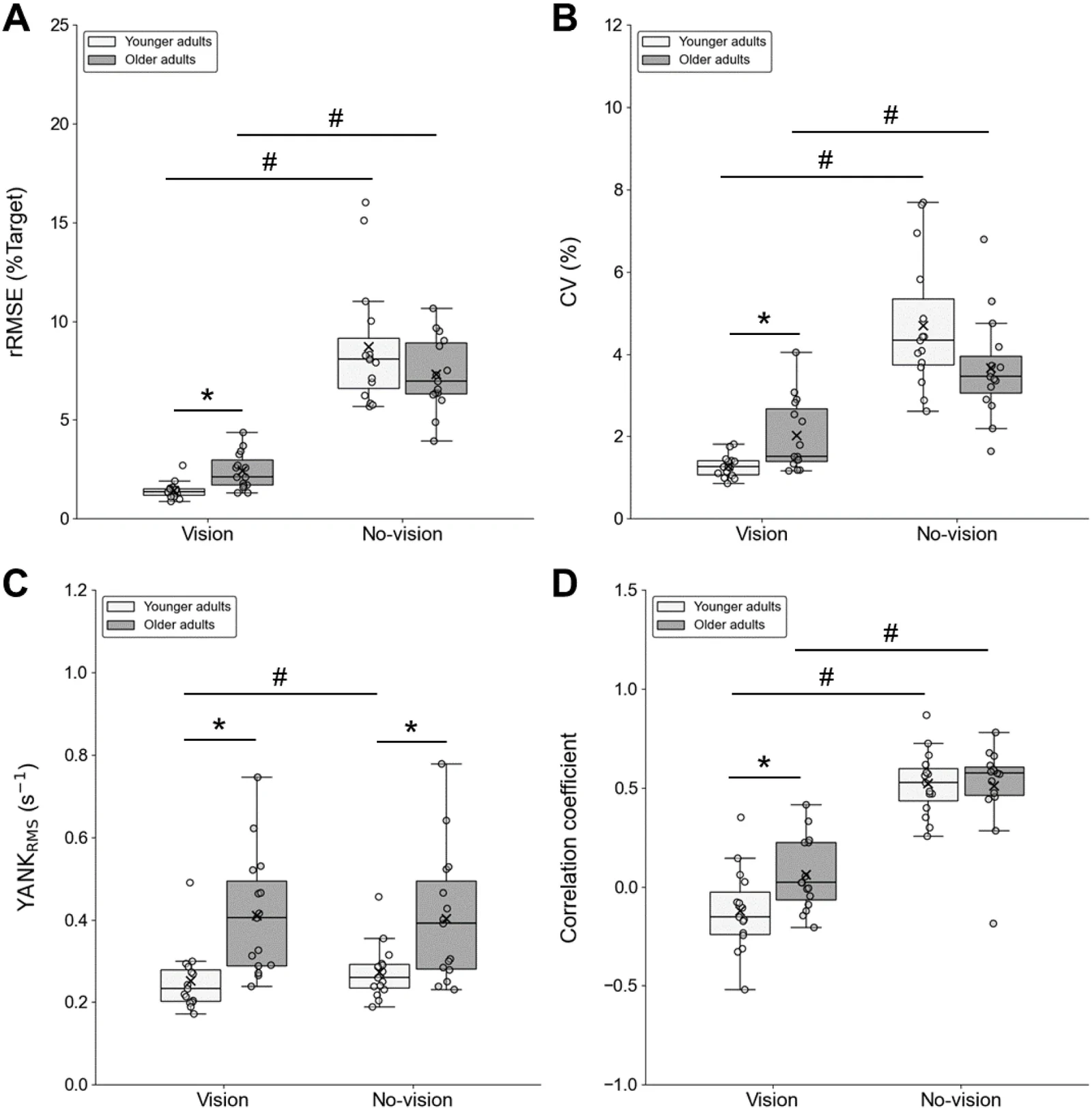
Bimanual force control performances and interlimb force coordination. (A) rRMSE showing a significant Group × Visual Condition interaction. (B) CV showing a significant Group × Visual Condition interaction. (C) YANK showing a significant Group × Visual Condition interaction. (D) Correlation coefficient showing a significant Group × Visual Condition interaction. Asterisk (*) indicates a significant difference between age groups. Number sign (#) denotes a significant difference between visual conditions. P < 0.05.

For force accuracy, a two-way mixed measures ANOVA on the rRMSE showed a significant Group × Visual Condition interaction [F (1, 28) = 8.429; P = 0.007; partial ƞ^2^ = 0.231; Figure 4A(Fig. 4)]. Specifically, the older group showed greater rRMSE values than the younger group in the vision condition (*P < *0.001). The no-vision condition significantly increased rRMSE values as compared with those in the vision condition for both younger (*P < *0.001) and older groups (*P < *0.001).

Regarding force variability, the analysis on the CV revealed a significant Group × Visual Condition interaction [*F *(1, 28) = 19.207; *P* < 0.001; partial ƞ^2^ = 0.407; Figure 4B(Fig. 4)]. Post-hoc analyses found higher CV values for the older group than the younger group in the vision condition (*P = *0.004). The values of CV in the no-vision condition were greater than those in the vision condition for both younger (*P < *0.001) and older groups (*P < *0.001).

For force smoothness, a two-way mixed measures ANOVA on the YANK found a significant Group × Visual Condition interaction [*F *(1, 28) = 9.735; *P* = 0.004; partial ƞ^2^ = 0.258; Figure 4C(Fig. 4)]. Specifically, the older group showed higher YANK values than the younger group for vision (*P = *0.001) and no-vision conditions (*P = *0.008), respectively. The younger group showed lower YANK values in the vision condition than those in the no-vision condition (*P = *0.003).

For interlimb force coordination, a two-way mixed measures ANOVA on the correlation coefficient revealed a significant Group × Visual Condition interaction [*F* (1, 28) = 5.402; *P* = 0.028; partial ƞ^2^ = 0.162; Figure 4D(Fig. 4)]. Specifically, the older group showed greater correlation coefficient values than the younger group in the vision condition (*P* = 0.019), whereas no significant group difference appeared in the no-vision condition (*P* = 0.850). Further, the no-vision condition significantly increased correlation coefficient values as compared with those in the vision condition for both younger (*P* < 0.001) and older groups (*P* < 0.001).

### ROC curve findings

The ROC curve analysis found significant bimanual force control differences between older and younger groups in the vision condition: (a) rRMSE (AUC = 0.867), (b) CV (AUC = 0.804), (c) YANK (AUC = 0.867), and (d) correlation coefficient (AUC = 0.747). In the no-vision condition, the analysis showed significant age-related force control differences between two groups in YANK (AUC = 0.771). Given that values of AUC greater than 0.7 indicate acceptable diagnostic accuracy, these four variables were good discriminators indicating bimanual force control deficits in older adults as compared with younger adults (Table 1(Tab. 1)).

### Correlations findings

**Figure 5:**
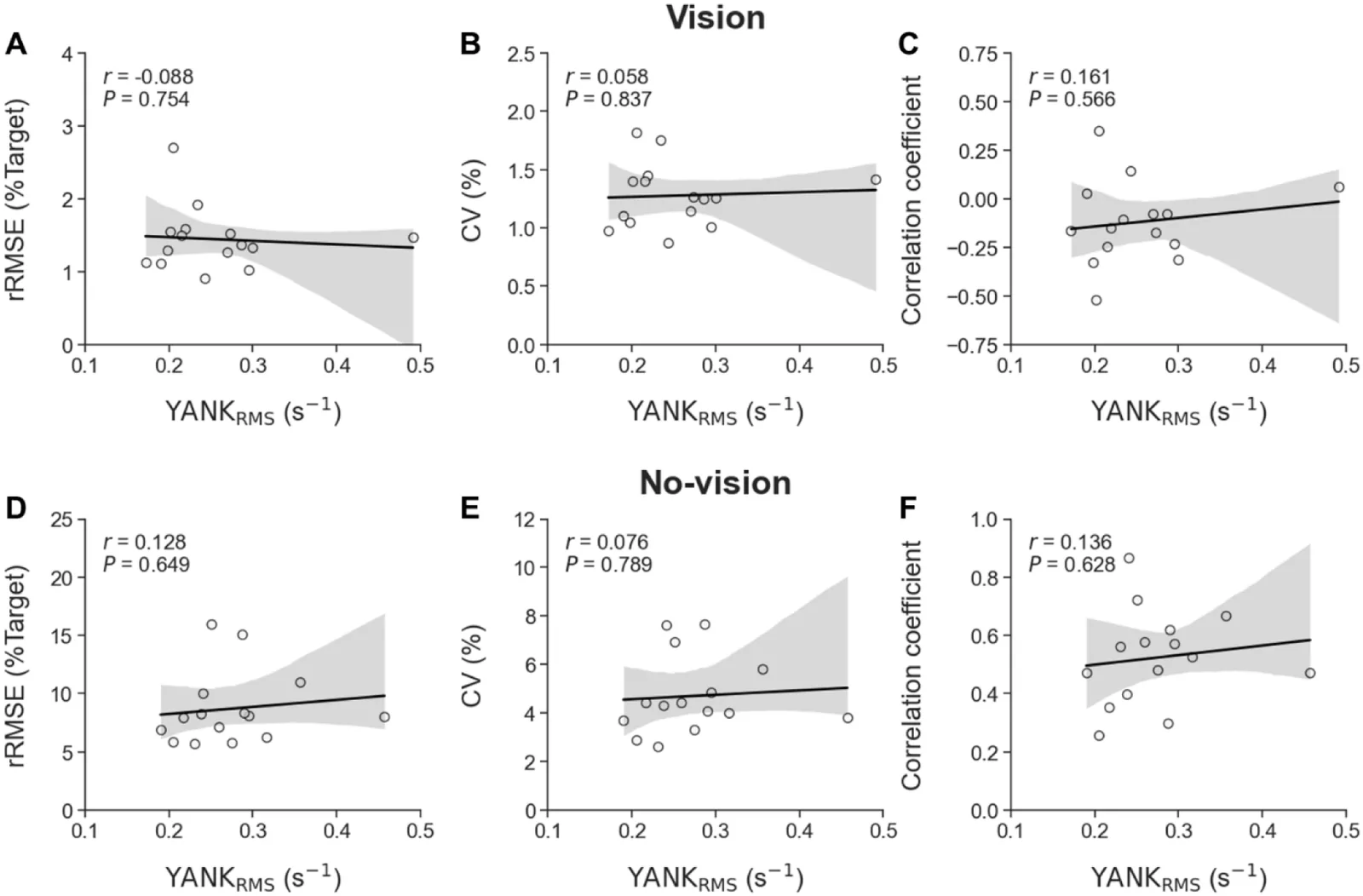
Correlations of YANK with conventional force control variables and interlimb force coordination for younger adults across visual conditions. (A) YANK vs. rRMSE in the vision condition. (B) YANK vs. CV in the vision condition. (C) YANK vs. correlation coefficient in the vision condition. (D) YANK vs. rRMSE in the no-vision condition. (E) YANK vs. CV in the no-vision condition. (F) YANK vs. correlation coefficient in the no-vision condition. Solid lines indicate linear regression fits, and shaded areas represent the 95 % confidence intervals.

**Figure 6:**
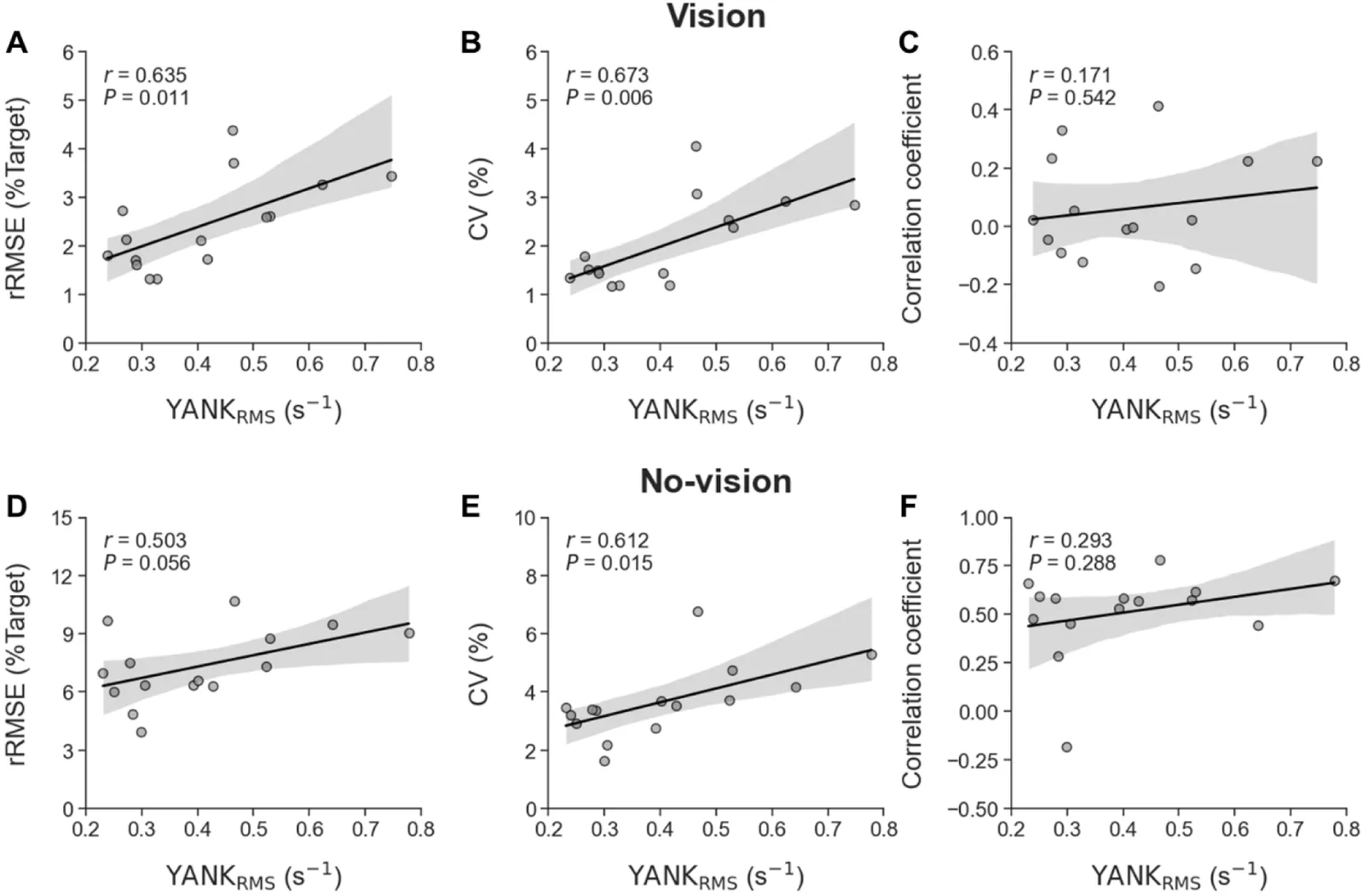
Correlations of YANK with conventional force control variables and interlimb force coordination for older adults across visual conditions. (A) YANK vs. rRMSE in the vision condition. (B) YANK vs. CV in the vision condition. (C) YANK vs. correlation coefficient in the vision condition. (D) YANK vs. rRMSE in the no-vision condition. (E) YANK vs. CV in the no-vision condition. (F) YANK vs. correlation coefficient in the no-vision condition. Solid lines indicate linear regression fits, and shaded areas represent the 95 % confidence intervals.

Pearson's correlation analyses showed no significant correlations between YANK and two conventional force control variables and interlimb force coordination including rRMSE, CV, and correlation coefficient across vision and no-vision conditions for the younger group (Figures 5A-5F(Fig. 5)). However, increased values of YANK for the older group at 10 % of MVC were significantly correlated with higher values of rRMSE (*r* = 0.635; *P* = 0.011; Figure 6A(Fig. 6)) and greater values of CV (*r* = 0.673; *P* = 0.006; Figure 6B(Fig. 6)) in the vision condition. In the no-vision condition, YANK was not significantly correlated with rRMSE (*r* = 0.503; *P* = 0.056; Figure 6D(Fig. 6)), whereas a significant positive correlation between YANK and CV was observed (*r* = 0.612; *P* = 0.015; Figure 6E(Fig. 6)). The analyses found no significant relationships between YANK and the correlation coefficient in the vision (Figure 6C(Fig. 6)) and no-vision conditions (Figure 6F(Fig. 6)) for the older group.

## Discussion

This study investigated force smoothness of older adults at a lower targeted level using YANK analysis. In the vision condition, older adults showed increased values of rRMSE and CV as compared with younger adults. Further, YANK values in older adults were higher than in younger adults across vision and no-vision conditions. The ROC curve analysis identified that rRMSE and YANK effectively discriminated changes in fine motor control capabilities between younger and older adults. Finally, older adults showed that increased values of YANK were related to greater values of rRMSE and CV in the vision condition, and a positive correlation between YANK and CV values was observed in the no-vision condition. However, these correlation patterns were not observed in younger adults. These findings support our hypothesis that aging would reduce force smoothness across the vision and no-vision conditions.

Consistent with prior findings (Galofaro et al., 2025[17]; Hu and Newell, 2011[25]; Jin et al., 2019[29]; Kang et al., 2022[30]), older adults showed greater task error, higher force variability, and impaired interlimb force coordination during bimanual force control tasks at 10 % of MVC with visual feedback. In addition to the conventional force control variables and interlimb force coordination, the YANK analysis revealed distinct characteristics of fine motor control in older adults, as indicated by decreased force smoothness across vision and no-vision conditions. To the best of our knowledge, these findings were the first to show age-induced impairments in force smoothness using the YANK approach. Yacoubi and Christou proposed that YANK captures the smoothness of the force trajectory, different from force variability quantified by the CV (Yacoubi and Christou, 2024[60]). Consistent with this hypothesis, our findings revealed that older adults exhibited increased YANK across both visual conditions compared with younger adults, whereas group differences in CV between age groups were not observed in the no-vision condition. Lin and colleagues assumed that high YANK values (i.e., lower force smoothness) indicate that the sensorimotor system drives rapid sensory feedback through sensory organs such as muscle spindles and Golgi tendon organs to facilitate reactive motor control (Binder-Macleod and Kesar, 2005[6]; De Groote et al., 2017[12]; Lin et al., 2019[39]). However, higher values of YANK in older adults were related to greater force error and variability in the present study, suggesting that increased YANK during fine motor control may reflect impaired temporal regulation of force output rather than an adaptive sensorimotor response (Pethick et al., 2022[45]; Sherman et al., 2024[51]). Further, younger adults showed lower YANK in the vision than the no-vision condition, indicating that visual feedback enabled smoother force output, whereas older adults failed to modulate force smoothness with visual feedback. Accordingly, less force smoothness in older adults should be reinterpreted through the lens of specific neurophysiological changes associated with aging (Hunter et al., 2016[27]; Seidler et al., 2010[50]).

Aging typically induces impairments in somatosensory and proprioceptive systems leading to excessive dependency on visual information (Barbanchon and Baudry, 2026[2]; Henry and Baudry, 2019[23]). For example, Tracy and colleagues reported that changes in variability from no-vision to vision conditions were significantly greater for older adults than younger adults while executing index finger force control at 2.5 % of MVC, indicating increased visual dependency in aging populations (Tracy et al., 2015[57]). Nevertheless, given that aging of the central nervous system often causes time delay in neural transmission during the sensorimotor feedback control loop, online visuomotor corrections may be compromised in older adults (Rasman et al., 2023[48]; Seidler et al., 2010[50]). To compensate for delayed error corrections, the brain may constantly generate excessive and rapid motor adjustment signals, disrupting the temporal smoothness of force trajectory (Li et al., 2023[37]). Moreover, aging often causes denervation of small and precise motor units and subsequent reinnervation by surviving larger motor units via axonal sprouting (Hepple and Rice, 2016[24]; Jang and Van Remmen, 2011[28]). This age-related neuromuscular remodeling induces increased overall size of motor unit indicating a greater number of muscle fibers innervated by a single motor neuron, influencing the precision of muscle contraction (Piasecki et al., 2018[46]). Considering fine force control at 10 % of MVC requiring precise recruitment of smaller motor units, the higher values of YANK in older adults suggest that these structural changes in the motor neuron pools may lead to coarse and discrete force adjustments rather than continuous and smooth modulation of motor outputs (Oomen and van Dieen, 2017[44]; Pethick et al., 2022[45]).

The ROC and correlation findings support that the levels of YANK during isometric force control tasks successfully distinguish age-induced impairments in fine motor control. In the vision condition, YANK showed higher diagnostic accuracy (AUC = 0.867) than CV (AUC = 0.804) in discriminating between groups. In the no-vision condition, only YANK revealed a significant group difference in the ANOVA and acceptable diagnostic accuracy (AUC = 0.771), whereas rRMSE, CV, and correlation coefficient failed to show significant differences between groups. These findings support a proposition that force smoothness captured by YANK reflected an aspect of motor control not fully represented by conventional variability measures (Kim et al., 2026[33]; Yacoubi and Christou, 2024[60]). Further, YANK might be sensitive to age-related decline even without visual feedback. Interestingly, the correlation results indicated that increased values of YANK were significantly related to more impairments in force control only in older adults for each vision condition, whereas no significant correlations appeared in younger adults. Previous studies often reported that submaximal force control error and variability increased at lower targeted levels (less than 10 % of MVC) (Ko et al., 2025[35]; Oomen and van Dieen, 2017[44]; Tracy, 2007[56]). These impairments during force control tasks requiring lower contraction intensity were crucial because most activities of daily living such as eating, dressing, and writing executed by older adults predominantly operate at forces below 20 % of MVC (Tikkanen et al., 2016[55]). Thus, the current findings suggest that age-related reduction in force smoothness in addition to impaired accuracy and variability may be notable motor control changes by compromised visuomotor and proprioceptive systems potentially interfering with activities of daily living (Seidler et al., 2010[50]). Taken together, aging potentially induces deficits in the neuromuscular system disrupting abilities to modulate error, variability, and temporal smoothness of motor outputs during fine motor tasks (Hunter et al., 2016[27]; Marmon et al., 2011[42]).

Despite lower temporal force smoothness patterns in older adults identified by the YANK approach, these findings should be carefully interpreted. First, older adults often failed to perform rapid and explosive motor corrections during gross motor tasks such as reactive balance control and fall prevention, inherently demanding sudden and moderate-to-high intensity force production (Oomen and van Dieen, 2017[44]). This means that aging may interfere with the bidirectional regulation of YANK, indicating an inability to suppress unintended force fluctuations during low-intensity fine motor control and a failure to generate sufficient YANK for rapid postural corrections (Bellumori et al., 2013[4]; Oomen and van Dieen, 2017[44]). Thus, future studies should investigate how the loss of force smoothness during precise and low-intensity force control tasks (e.g., ankle force control) is linked with a failure to adequately modulate YANK values in gross motor tasks requiring moderate-to-high force intensities in older adults. Second, although we suggested potential neurophysiological mechanisms underlying age-related force smoothness, investigating how the central and peripheral nervous systems are associated with YANK regulation strategies in aging populations is necessary. For example, decoding cortical drive and motor unit behaviors related to changes in YANK may elucidate neuromuscular rehabilitation targets for improving fine motor control in older adults.

In conclusion, the current study identified that older adults revealed decreased force smoothness during bimanual force control across vision and no-vision conditions at 10 % of MVC. Moreover, the YANK approach effectively distinguished fine motor control deficits in older adults from younger adults, and this was significantly related to impaired conventional force control performances including less force accuracy and greater force variability. These findings suggest that the YANK analysis can be used for evaluating age-related impairments in the neuromuscular system. Further, considering potential relationship between pathological force oscillations and force smoothness, future studies should explore whether YANK can serve as an effective assessment for the early identification of neuromuscular deficits in patients with neurological diseases such as Parkinson's disease.

## Declaration

### CRediT authorship contribution statement

Hajun Lee: Data curation, Software, Investigation, Visualization, Writing - original draft, Writing - review & editing. Hanall Lee: Data curation. Jinwon Shin: Writing - original draft. Eo-Jin Son: Writing - original draft. Nyeonju Kang: Conceptualization, Resources, Supervision, Funding acquisition, Validation, Methodology, Project administration, Writing - review & editing.

### Conflict of interest

The authors declare that they have no conflict of interest.

### Funding

This work was supported by Incheon National University Research Grant in 2026 (2026-0006) to NK.

### Artificial Intelligence (AI) - assisted technology

Artificial intelligence was not used in the preparation, writing, and editing of this manuscript.

## Figures and Tables

**Table 1:**
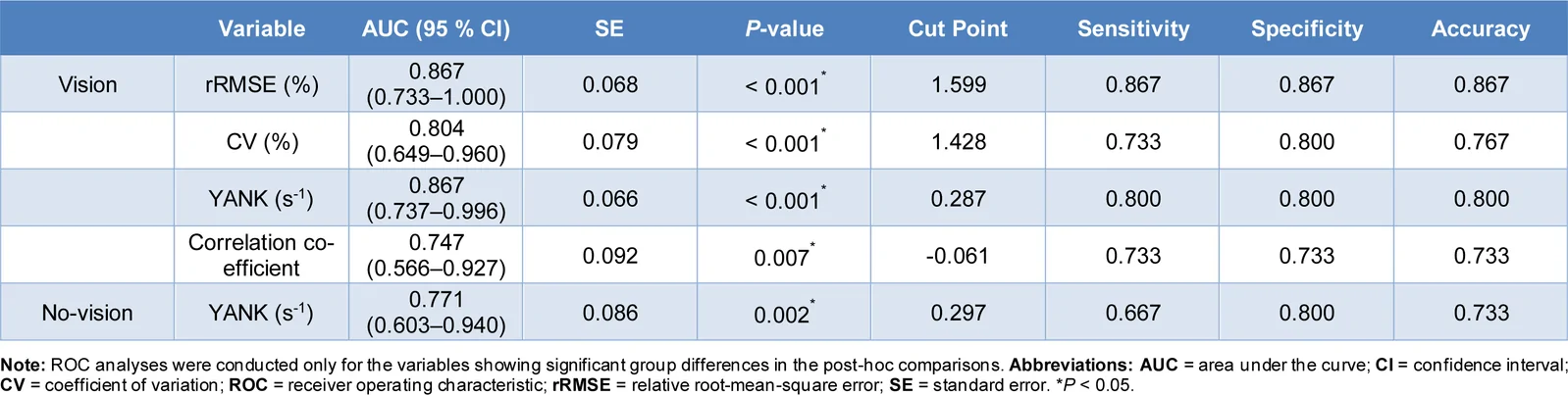
ROC curve analysis on force control variables and interlimb force coordination between younger and older adults

**Figure 1:**
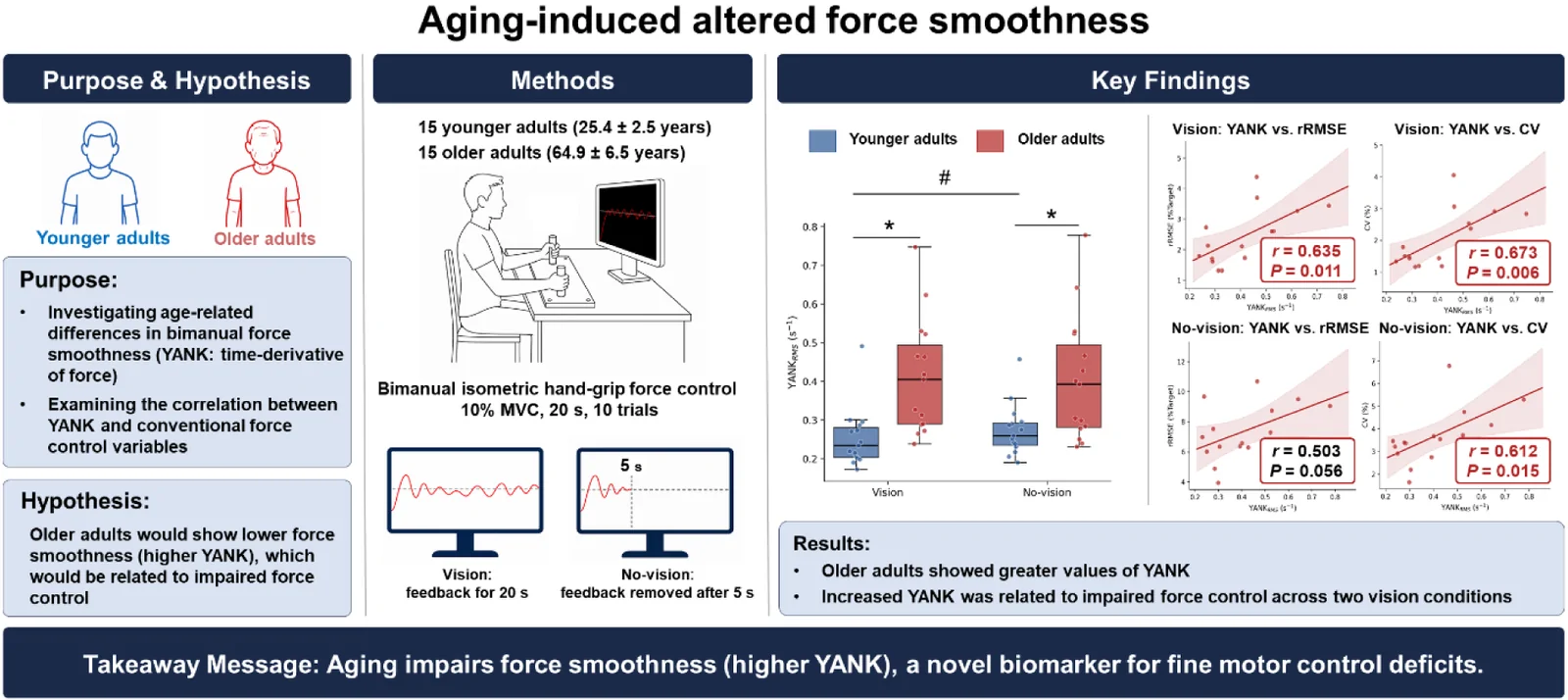
Graphical abstract

## References

[1] Ballardini G, Ponassi V, Galofaro E, Carlini G, Marini F, Pellegrino L (2019). Interaction between position sense and force control in bimanual tasks. J Neuroeng Rehabil.

[2] Barbanchon C, Baudry S (2026). Aging increases visual dependency and disrupts sensory strategies in upright standing. GeroScience.

[3] Bayle N, Lempereur M, Hutin E, Motavasseli D, Remy-Neris O, Gracies JM (2023). Comparison of various smoothness metrics for upper limb movements in middle-aged healthy subjects. Sensors (Basel).

[4] Bellumori M, Jaric S, Knight CA (2013). Age-related decline in the rate of force development scaling factor. Motor Control.

[5] Billot M, Calvani R, Urtamo A, Sánchez-Sánchez JL, Ciccolari-Micaldi C, Chang M (2020). Preserving mobility in older adults with physical frailty and sarcopenia: opportunities, challenges, and recommendations for physical activity interventions. Clin Interv Aging.

[6] Binder-Macleod S, Kesar T (2005). Catchlike property of skeletal muscle: recent findings and clinical implications. Muscle Nerve.

[7] Cabeza R, Anderson ND, Locantore JK, McIntosh AR (2002). Aging gracefully: compensatory brain activity in high-performing older adults. Neuroimage.

[8] Camacho-Villa MA, Giráldez-García MA, Sevilla-Sanchez M, Rivera-Mejía SL, Carballeira E (2025). Relationship between force steadiness and functionality in older adults: a systematic review with meta-analysis. Scand J Med Sci Sports.

[9] Carr JC (2024). A steady interest of analytical approaches to quantify skeletal muscle force control. J Appl Physiol.

[10] Christou EA (2011). Aging and variability of voluntary contractions. Exerc Sport Sci Rev.

[11] Critchley K, Kokubu M, Iemitsu M, Fujita S, Isaka T (2014). Age-related differences in the availability of visual feedback during bimanual pinch. Eur J Appl Physiol.

[12] De Groote F, Allen JL, Ting LH (2017). Contribution of muscle short-range stiffness to initial changes in joint kinetics and kinematics during perturbations to standing balance: a simulation study. J Biomech.

[13] Delvenne JF, Malloy E (2025). Functional implications of age-related atrophy of the corpus callosum. Neurosci Biobehav Rev.

[14] Djafari Y, Arshi AR, Rajabi H (2025). Hierarchical clustering approach to movement smoothness and temporal adaptation in rhythmic step aerobics training in middle aged women. Sci Rep.

[15] Enoka RM, Farina D (2021). Force steadiness: from motor units to voluntary actions. Physiology (Bethesda).

[16] Faul F, Erdfelder E, Lang AG, Buchner A (2007). G*Power 3: a flexible statistical power analysis program for the social, behavioral, and biomedical sciences. Behav Res Methods.

[17] Galofaro E, Valè N, Ballardini G, Smania N, Casadio M (2025). Age-related differences in bimanual isometric force tracking. IEEE Trans Neural Syst Rehabil Eng.

[18] Goble DJ, Coxon JP, Van Impe A, De Vos J, Wenderoth N, Swinnen SP (2010). The neural control of bimanual movements in the elderly: brain regions exhibiting age-related increases in activity, frequency-induced neural modulation, and task-specific compensatory recruitment. Hum Brain Mapp.

[19] Godde B, Trautmann M, Erhard P, Voelcker-Rehage C (2018). Motor practice in a force modulation task in young and middle-aged adults. J Electromyogr Kinesiol.

[20] Gulde P, Hermsdörfer J (2018). Smoothness metrics in complex movement tasks. Front Neurol.

[21] Hanley JA, McNeil BJ (1982). The meaning and use of the area under a receiver operating characteristic (ROC) curve. Radiology.

[22] He H, Luo C, Chang X, Shan Y, Cao W, Gong J (2017). The functional integration in the sensory-motor system predicts aging in healthy older adults. Front Aging Neurosci.

[23] Henry M, Baudry S (2019). Age-related changes in leg proprioception: implications for postural control. J Neurophysiol.

[24] Hepple RT, Rice CL (2016). Innervation and neuromuscular control in ageing skeletal muscle. J Physiol.

[25] Hu X, Newell KM (2011). Aging, visual information, and adaptation to task asymmetry in bimanual force coordination. J Appl Physiol.

[26] Hu X, Newell KM (2011). Visual information gain and task asymmetry interact in bimanual force coordination and control. Exp Brain Res.

[27] Hunter SK, Pereira HM, Keenan KG (2016). The aging neuromuscular system and motor performance. J Appl Physiol.

[28] Jang YC, Van Remmen H (2011). Age-associated alterations of the neuromuscular junction. Exp Gerontol.

[29] Jin Y, Seong J, Cho Y, Yoon B (2019). Effects of aging on motor control strategies during bimanual isometric force control. Adapt Behav.

[30] Kang N, Ko DK, Cauraugh JH (2022). Bimanual motor impairments in older adults: an updated systematic review and meta-analysis. EXCLI J.

[31] Kang N, Roberts LM, Aziz C, Cauraugh JH (2019). Age-related deficits in bilateral motor synergies and force coordination. BMC Geriatr.

[32] Kenway LC, Bisset LM, Kavanagh JJ (2016). Visually guided targeting enhances bilateral force variability in healthy older adults. Neurobiol Aging.

[33] Kim JJ, Shankar VR, Delmas S, Choi YJ, Weintraub M, Malik RJ (2026). Distinct effects of visual gain manipulation on force variability and smoothness during constant isometric contractions. J Neurophysiol.

[34] Ko DK, Kang N (2024). Aging impairs unimanual and bimanual hand-grip force control capabilities. Appl Sci.

[35] Ko DK, Lee H, Lee H, Kang N (2025). Bilateral ankle dorsiflexion force control impairments in older adults. PLoS One.

[36] Lasko TA, Bhagwat JG, Zou KH, Ohno-Machado L (2005). The use of receiver operating characteristic curves in biomedical informatics. J Biomed Inform.

[37] Li N, Liu J, Xie Y, Ji W, Chen Z (2023). Age-related decline of online visuomotor adaptation: a combined effect of deteriorations of motor anticipation and execution. Front Aging Neurosci.

[38] Lin CH, Sung WH, Chiang SL, Lee SC, Lu LH, Wang PC (2019). Influence of aging and visual feedback on the stability of hand grip control in elderly adults. Exp Gerontol.

[39] Lin DC, McGowan CP, Blum KP, Ting LH (2019). Yank: the time derivative of force is an important biomechanical variable in sensorimotor systems. J Exp Biol.

[40] Lodha N, Moon H, Kim C, Onushko T, Christou EA (2016). Motor output variability impairs driving ability in older adults. J Gerontol A Biol Sci Med Sci.

[41] Maes C, Gooijers J, Orban de Xivry JJ, Swinnen SP, Boisgontier MP (2017). Two hands, one brain, and aging. Neurosci Biobehav Rev.

[42] Marmon AR, Pascoe MA, Schwartz RS, Enoka RM (2011). Associations among strength, steadiness, and hand function across the adult life span. Med Sci Sports Exerc.

[43] Morrison S, Sosnoff JJ, Heffernan KS, Jae SY, Fernhall B (2013). Aging, hypertension and physiological tremor: the contribution of the cardioballistic impulse to tremorgenesis in older adults. J Neurol Sci.

[44] Oomen NM, van Dieen JH (2017). Effects of age on force steadiness: a literature review and meta-analysis. Ageing Res Rev.

[45] Pethick J, Taylor MJ, Harridge SD (2022). Aging and skeletal muscle force control: current perspectives and future directions. Scand J Med Sci Sports.

[46] Piasecki M, Ireland A, Piasecki J, Stashuk DW, Swiecicka A, Rutter MK (2018). Failure to expand the motor unit size to compensate for declining motor unit numbers distinguishes sarcopenic from non-sarcopenic older men. J Physiol.

[47] Qiu F, Liu X, Chen C (2025). Effects of aging on motor unit properties in isometric elbow flexion. Bioengineering (Basel).

[48] Rasman BG, van der Zalm C, Forbes PA (2023). Age-related impairments and influence of visual feedback when learning to stand with unexpected sensorimotor delays. Front Aging Neurosci.

[49] Rattanawan P (2022). Correlations between hand dexterity and bimanual coordination on the activities of daily living in older adults with mild cognitive impairment. Dement Geriatr Cogn Dis Extra.

[50] Seidler RD, Bernard JA, Burutolu TB, Fling BW, Gordon MT, Gwin JT (2010). Motor control and aging: links to age-related brain structural, functional, and biochemical effects. Neurosci Biobehav Rev.

[51] Sherman DA, Darendeli A, Soto O, Tunik E, Stefanik JJ, Yarossi M (2024). Beyond force steadiness: potential challenges in measuring smoothness of force through yank. J Appl Physiol.

[52] Shizuka Y, Sawai S, Nishida T, Fujikawa S, Yamamoto R, Shimizu N, Nakano H (2025). Age-related changes in upper limb motor function, focusing on bimanual coordination. Physical Therapy and Rehabilitation - Research and Clinical Application.

[53] Soto O, Cros D (2011). Direct corticospinal control of force derivative. J Neurosci.

[54] Strote C, Gölz C, Stroehlein JK, Haase FK, Koester D, Reinsberger C (2020). Effects of force level and task difficulty on force control performance in elderly people. Exp Brain Res.

[55] Tikkanen O, Sipila S, Kuula AS, Pesola A, Haakana P, Finni T (2016). Muscle activity during daily life in the older people. Aging Clin Exp Res.

[56] Tracy BL (2007). Force control is impaired in the ankle plantarflexors of elderly adults. Eur J Appl Physiol.

[57] Tracy BL, Hitchcock LN, Welsh SJ, Paxton RJ, Feldman-Kothe CE (2015). Visuomotor correction is a robust contributor to force variability during index finger abduction by older adults. Front Aging Neurosci.

[58] Vieluf S, Sleimen-Malkoun R, Voelcker-Rehage C, Jirsa V, Reuter EM, Godde B (2017). Dynamical signatures of isometric force control as a function of age, expertise, and task constraints. J Neurophysiol.

[59] Yacoubi B, Christou EA (2024). Last word on viewpoint: rethinking force steadiness: a new perspective—yank magnitude: a metric of unsteadiness during the constant isometric force task. J Appl Physiol.

[60] Yacoubi B, Christou EA (2024). Rethinking force steadiness: a new perspective. J Appl Physiol.

[61] Yan JH (2000). Effects of aging on linear and curvilinear aiming arm movements. Exp Aging Res.

